# Challenges in the Management of a Difficult-to-Treat Patient With Hairy Cell Leukemia: A Case Report

**DOI:** 10.7759/cureus.76594

**Published:** 2024-12-29

**Authors:** Bryan Valdivia, Pablo Perez, Alvaro Cortez

**Affiliations:** 1 Internal Medicine, Saint Joseph Clinic PC, Dalton, USA; 2 Family Medicine, Augusta University Medical College of Georgia, Chatsworth, USA

**Keywords:** braf inhibitors, btk inhibitor, cladribine, hairy cell, hairy cell leukemia (hcl), hematologic malignancy, leukemia, rituximab

## Abstract

Hairy cell leukemia (HCL) is a rare, chronic B-cell malignancy with an indolent course that typically responds well to purine nucleoside analogs, such as cladribine. We present the case of a 74-year-old woman with nearly three decades of recurrent HCL, marked by multiple relapses and significant toxicities to various treatments, including purine analogs, BRAF inhibitors, BTK inhibitors, a cytoreductive agent, and the monoclonal antibody rituximab. Despite severe allergic reactions and intolerances to standard therapies, the patient achieved multiple remissions. This case underscores the challenges in managing treatment-resistant HCL and highlights the need for personalized treatment strategies in patients with relapsed disease and drug toxicities.

## Introduction

Hairy cell leukemia (HCL) is a rare chronic B-cell malignancy that affects the bone marrow, peripheral blood, and the spleen. It represents 2% of lymphoid leukemias, and the median age of diagnosis is 55 years old, with approximately 900 new cases of HCL every year in the United States [1]. Patients usually present with non-specific symptoms related to cytopenias, such as fatigue and weakness. However, other clinical findings, such as abdominal pain, can be present. On a physical exam, lymphadenopathy and splenomegaly may be observed, although these are usually late findings [2].

The diagnosis of HCL includes studies on peripheral blood showing anemia and thrombocytopenia, flow cytometry showing markers positive for CD19, CD20, CD22, CD123, and CD103, and peripheral smear showing lymphocytes with hair-like cytoplasm projections, termed "hairy cells," that stain positive for tartrate-resistant acid phosphatase (TRAP); bone marrow aspiration is also used as a diagnostic tool where a "dry tap" can be found, meaning an inability to perform the aspiration due to the marrow fibrosis induced by the hairy cells [3,4].

The goal of treatment for HCL is to achieve a complete remission, which is defined as the following: (1) the absence of hairy cells in the blood and marrow; (2) the liver, lymph nodes, and spleen are of normal size; and (3) blood cell and marrow cell counts have returned to normal. Splenectomy is usually considered in patients with abdominal pain due to massive splenomegaly and severe pancytopenia due to spleen sequestration. Nevertheless, cladribine (2-chlorodeoxyadenosine), a purine analog, is usually the first-line medication to treat HCL due to its effectiveness and less toxicity; in patients with a normal renal function, the therapy should consist of a standard regimen of an intravenous infusion of 0.1 mg/kg per day for seven days, or 0.14 mg/kg/day intravenously over two hours once per day for five days, or 0.1-0.14 mg/kg/day subcutaneously once per day for five days. It has been shown that cladribine induces a complete response in 90% of patients after one cycle (five to seven days). However, a small percentage of patients show a relapse of the disease, requiring treatment with another purine analog, such as pentostatin at a dose of 4 mg/m^2 ^intravenously once every two weeks, or another medication combination, such as cladribine with rituximab maintenance [5].

In refractory or progressive cases, fludarabine alone or in combination with rituximab can be used in a dose of fludarabine 40 mg/m^2^ per day orally on days one to five adjusted for renal function (50% dose reduction for creatinine clearance 30-70 mL/minute; not used if creatinine clearance < 30 mL/minute) and rituximab 375 mg/m^2^ intravenously on day one administered every 28 days for four cycles [6]. Other options include bendamustine and interferon-alpha, and in cases of *BRAF* mutation in HCL, the BRAF inhibitor vemurafenib has been shown to be efficacious in both relapsed and refractory cases [7]. A study indicates an alternative treatment for HCL that is refractory to vemurafenib plus rituximab, involving a combination of venetoclax with or without rituximab, which may serve as a safe and effective salvage option following the failure of the aforementioned medication combination [8].

This is a case report of a female patient with recurrent HCL who had adverse responses to multiple cancer therapies.

## Case presentation

This case involves a 74-year-old Caucasian woman with a past medical history of HCL, hypertension, chronic diastolic heart failure, thyroid cancer status post-partial thyroidectomy, and vitamin D deficiency. The patient was diagnosed with HCL in 1996 at the age of 46. She received a cycle of cladribine, resulting in an excellent response and complete remission. However, five years later, in 2001, she experienced a relapse. At that time, the treatment consisted of another cycle of cladribine and a splenectomy. She subsequently achieved remission and remained asymptomatic until 2015, when a second relapse occurred. The patient was treated with a combination of fludarabine and rituximab, but these therapies were poorly tolerated. Rituximab induced a severe allergic reaction characterized by hives, while fludarabine caused significant myelosuppression, requiring multiple blood transfusions. As a result, her regimen was changed to three months of pentostatin, leading to remission.

In December 2019, during a routine oncological evaluation, the patient was found to have increasing WBC counts and declining hemoglobin levels. A bone marrow biopsy revealed 90% hypercellular marrow with hairy cell infiltration. She was started on pentostatin 4 mg/m^2^ every two weeks in February 2020, completing six cycles. Despite treatment, residual disease was evident, with 2% of lymphocytes remaining positive for hairy cells by April 2020. By May 2020, her laboratory parameters, including WBC count, hemoglobin, and platelet levels, demonstrated improvement, as shown in Table 1. The patient did not attend follow-up appointments until January 2021, when laboratory results revealed an elevated WBC count of 19 × 10^3^/µL. Flow cytometry confirmed 48% hairy cell involvement, although hemoglobin and platelet counts remained normal.

**Table 1:** Laboratory analyses WBC: white blood cell

Parameter	Values	Units	Reference Values
WBC count	4.7 x 10^3^	µL	4.8-10.8 x 10^3^
Hemoglobin	9	g/dL	12-16
Platelet count	400 x 10^3^	µL	130-400 x 10^3^

A repeat bone marrow biopsy in February 2021 demonstrated 100% bone marrow involvement with hairy cells. Karyotyping identified a *BRAF* mutation. She was initiated on vemurafenib (Zelboraf); however, after seven days of treatment, it was discontinued due to severe adverse effects, including diarrhea, fatigue, and facial rash; at this time, a repeat CBC showed a decrease in WBC count (Table 2).

**Table 2:** Laboratory analyses after treatment with vemurafenib WBC: white blood cell

Parameter	Values	Units	Reference Values
WBC count	8.9 x 10^3^	µL	4.8-10.8 x 10^3^

A subsequent trial of encorafenib was also abandoned due to a severe allergic reaction involving shortness of breath and rash, requiring emergency care. At this point, her oncologist informed her of a recent study showing an 87% complete response rate at three years for relapsed refractory HCL patients with a drug combination of BRAF inhibitor with rituximab; however, due to her previous life-threatening allergic reaction to rituximab, she was not eligible for this medication combination. In mid-2021, the patient was referred to Vanderbilt University Medical Center, where she underwent a third course of cladribine. Post-treatment, her WBC count and absolute lymphocyte count (ALC) improved, as shown in Table 3. Rituximab was proposed but declined due to her prior life-threatening allergic reaction. She opted for close monitoring.

**Table 3:** Laboratory analyses after the second course of cladribine treatment WBC: white blood cell

Parameter	Values	Units	Reference Values
WBC count	3.3 x 10^3^	µL	4.8-10.8 x 10^3^
Absolute lymphocyte count	0.3 x 10^3^	µL	1-4.8 x 10^3^

By August 2022, her WBC count increased to 11.2 × 10^3^/µL, and she reported pruritus and a prickly sensation under her skin. Flow cytometry confirmed the presence of active disease. Although rituximab was again suggested, the patient refused this treatment. By January 2023, she initiated therapy with ibrutinib, starting with one pill daily and titrating up to two pills daily. However, she experienced severe fatigue, nausea, and anorexia, leading to treatment discontinuation in the third week. In February 2023, the patient sought consultation at the National Cancer Institute. Her hemoglobin and platelet counts remained normal at this time, but her WBC count has increased to 54 × 10^3^/µL. She was prescribed acalabrutinib 100 mg BID with plans to introduce venetoclax after four weeks. However, the patient developed extreme weakness and lightheadedness during the first week, prompting dose reduction and eventual discontinuation in May 2023. At this time, she opted not to pursue any further treatment, and after a month, her WBC count increased, while her platelet count and hemoglobin levels were low, as shown in Table 4.

**Table 4:** Laboratory analyses in June 2023 WBC: white blood cell

Parameter	Values	Units	Reference Values
WBC count	95 x 10^3^	µL	4.8-10.8 x 10^3^
Hemoglobin	11.7	g/dL	12-16
Platelet count	129 x 10^3^	µL	130-400 x 10^3^

By mid-2023, disease progression was evident, with worsening cytopenias and an increasing WBC count. Hydroxyurea 500 mg daily was initiated to control the WBC count, but it was discontinued after a single dose due to severe pruritus and a crawling sensation. Attempts to restart hydroxyurea at a lower dose were unsuccessful due to insurance issues. Two months later, she consented to a follow-up at Vanderbilt again due to her increasing white blood cell count and declining hemoglobin and platelet levels, as indicated in Table 5. In October 2023, she was started on cladribine for the third time. She was admitted to Hamilton Regional Medical Center for cladribine treatment, and she exhibited commendable tolerance to the five-day treatment, showing no indications of tumor lysis syndrome. Her blood count recovered over the next three months, and she continued with follow-ups every three to four months. Additionally, she expressed that she will not consider further treatment unless her laboratory results deteriorate significantly.

**Table 5:** Laboratory analyses before the third course of cladribine treatment WBC: white blood cell

Parameter	Values	Units	Reference Values
WBC count	124 x 10^3^	µL	4.8-10.8 x 10^3^
Hemoglobin	11.6	g/dL	12-16
Platelet count	101 x 10^3^	µL	130-400 x 10^3^

She was seen in April 2024 with a normal WBC count, and her hemoglobin and platelet levels were completely stable; she did not report any complaints at that time. The last time she was seen in August 2024, she was asymptomatic, and labs showed a slight increase in WBC count and ALC, with normal levels of hemoglobin and platelets, as shown in Table 6. She reported no fever, weight loss, shortness of breath, or nausea/vomiting. As noted, her WBC count is slowly increasing, and she will be reassessed in four months with no active treatment unless she has a significant progression in the future.

**Table 6:** Laboratory analyses in April and August 2024 WBC: white blood cell

Parameter	Values in April 2024	Values in August 2024	Units	Reference Values
WBC count	6.2 x 10^3^	8.4 x 10^3^	µL	4.8-10.8 x 10^3^
Absolute lymphocyte count	0.8 x 10^3^	1.7 x 10^3^	µL	1-4.8 x 10^3^
Hemoglobin	13.2	14.3	g/dL	12-16
Platelet count	250 x 10^3^	211 x 10^3^	µL	130-400 x 10^3^

## Discussion

This case report describes a 74-year-old woman with relapsed HCL over nearly three decades and shows the importance and challenges in long-term management and individualized treatment approaches. HCL is a rare, indolent B-cell malignancy that accounts for approximately 2% of all leukemias and is highly responsive to cladribine. Relapses can occur several years later, as observed in this patient, and they can receive various alternative therapies; however, her multiple intolerances to standard therapies, including rituximab, fludarabine, BRAF inhibitors, and BCL-2 inhibitors, make this case unique. A key distinguishing feature of this case is the patient's severe allergic reactions and side effects to multiple treatments, which limited different therapeutic options.

The combination of purine analogs, such as cladribine, plus rituximab maintenance has been studied and used in patients with relapsed HCL. Andrasiak et al. found great results using this cladribine followed by rituximab maintenance for patients newly diagnosed or relapsed after one prior therapy [9]. A phase II study by Chihara et al. showed that 94% of 68 patients who received concurrent therapy of cladribine with rituximab were free of MRD (measurable residual disease) [10]. However, our patient had a life-threatening hypersensitivity reaction to rituximab, which sharply contrasts with the experiences documented in other studies where rituximab is well tolerated and significantly improves outcomes [11,12]. The absence of rituximab tolerance in our case required exploration of alternative agents, none of which were well tolerated in this patient.

BRAF inhibitors such as vemurafenib and encorafenib have been an important advancement in the management of relapsed/refractory HCL due to the common *BRAF* *V600E* mutation in nearly all patients [13]. In this case, the rapid reduction in WBC count with vemurafenib was promising, aligning with outcomes seen in other studies where BRAF inhibitors for HCL were used. The literature for the use of vemurafenib in the treatment of relapsed HCL shows good results; two multicenter studies were performed in Italy and the United States, showing that 91% of patients (n = 54) had an overall response while 35% of them had a complete response in the intention-to-treat analysis [14-16]. However, the patient in this report showed adverse effects, including severe fatigue, rash, and gastrointestinal issues, which led to early discontinuation.

The use of BTK inhibitors, such as ibrutinib and acalabrutinib, provided another option for relapsed HCL, but as with previous treatments, this patient did not tolerate these agents due to severe side effects. A phase II study on ibrutinib in HCL showed an estimated 60-month PFS (progression-free survival) of 68%, which makes it a good option for those who are not expected to benefit from purine analogs [17]. There are no studies in the literature regarding the use of acalabrutinib for relapsed HCL; however, due to its reduced side effects and quicker absorption than ibrutinib, it can be considered in a group of patients who have a refractory/relapsed HCL [18,19].

The biggest challenge in the management of this patient was her inability to tolerate many of the available therapies, which limited many combination therapies that have been shown to improve survival in relapsed HCL. Additionally, this patient refused further rituximab treatment due to her prior life-threatening allergic reactions, which limited treatment options, especially when rituximab is part of many combination therapies. Desensitization protocols might have been beneficial for this patient; however, the patient was reluctant to attempt rituximab again [20,21].

This patient exhibited a favorable response to cladribine therapy for the third time, resulting in the normalization of her hemoglobin, WBC count, and platelet levels. Currently, 10 months after her last therapy with cladribine, she is showing a gradual increase in WBC count without any clinical symptoms. She will undergo reassessment in four months and will receive treatment only if there is significant progression.

## Conclusions

This case highlights the critical role of personalized treatment strategies in managing relapsed or refractory HCL, particularly in patients with multiple treatment-related toxicities. It underscores the necessity of balancing efficacy with tolerability when selecting therapies and emphasizes the importance of patient-centered care, including thorough communication about available options and potential risks. Furthermore, this case illustrates the pressing need to develop more tolerable therapeutic approaches for this challenging patient population.
